# PD-1 and ICOS counter-regulate tissue resident regulatory T cell development and IL-10 production during flu

**DOI:** 10.3389/fimmu.2022.984476

**Published:** 2022-09-08

**Authors:** Michael C. McGee, Tianyi Zhang, Nicholas Magazine, Rezwanul Islam, Mariano Carossino, Weishan Huang

**Affiliations:** ^1^ Department of Pathobiological Sciences, School of Veterinary Medicine, Louisiana State University, Baton Rouge, LA, United States; ^2^ Department of Microbiology and Immunology, College of Veterinary Medicine, Cornell University, Ithaca, NY, United States

**Keywords:** influenza infections, tissue resident T cells, Foxp3, ICOS, PD-1, IL-10

## Abstract

Regulatory T cells that express the transcription factor Foxp3 (Treg cells) are a highly heterogenous population of immunoregulatory cells critical for maintaining immune homeostasis and preventing immunopathology during infections. Tissue resident Treg (TR-Treg) cells are maintained within nonlymphoid tissues and have been shown to suppress proinflammatory tissue resident T cell responses and promote tissue repair. Human populations are repetitively exposed to influenza infections and lung tissue resident effector T cell responses are associated with flu-induced long-term pulmonary sequelae. The kinetics of TR-Treg cell development and molecular features of TR-Treg cells during repeated and/or long-term flu infections are unclear. Utilizing a Foxp3^RFP^/IL-10^GFP^ dual reporter mouse model along with intravascular fluorescent *in vivo* labeling, we characterized the TR-Treg cell responses to repetitive heterosubtypic influenza infections. We found lung tissue resident Treg cells accumulated and expressed high levels of co-inhibitory and co-stimulatory receptors post primary and secondary infections. Blockade of PD-1 or ICOS signaling reveals that PD-1 and ICOS signaling pathways counter-regulate TR-Treg cell expansion and IL-10 production, during secondary influenza infection. Furthermore, the virus-specific TR-Treg cell response displayed distinct kinetics, when compared to conventional CD4^+^ tissue resident memory T cells, during secondary flu infection. Our results provide insight into the tissue resident Foxp3^+^ regulatory T cell response during repetitive flu infections, which may be applicable to other respiratory infectious diseases such as tuberculosis and COVID.

## Introduction

Foxp3^+^ regulatory T (Treg) cells are a population of immunomodulatory CD4^+^ T cells critical in preventing autoimmunity, immunopathology, and maintaining immune homeostasis (1–3). Treg cells exhibit great heterogeneity and may be found in circulation and secondary lymphoid organs (SLO) (4). Tissue resident Treg cells are maintained in nonlymphoid tissue (NLT) and barrier sites under steady conditions and display limited or no recirculation (4–8). The importance of tissue resident Treg cells extends beyond immunosuppressive functions. For example, a well-described tissue resident Treg cell population in visceral adipose tissue (VAT) can preserve insulin sensitivity and regulate adipocyte metabolism (9–11), while Treg cells accumulate and promote tissue repair upon muscle injury (12).

CD4^+^ and CD8^+^ tissue resident memory (Trm) cells are present at barrier sites and provide a rapid response to infection (13–17). However, Trm cells have also been found to be drivers of disease and immunopathology (18–22). Recent studies have probed interactions between Treg cells and Trm cells. In the intestines, Treg cells have been shown to promote CD8^+^ Trm cell development, but suppress the CD8^+^ Trm cell response leading to development of viral sequelae in the lungs (23, 24). Tissue resident Treg cells have been shown to limit immunopathology driven by CD4^+^ Trm cells in a chronic *Aspergillus fumigatus* model (18). Therefore, understanding the tissue resident Treg cell responses may provide insight into vaccine development and therapeutics for tissue repair, chronic inflammation, and viral sequelae.

Influenza infection is a leading cause of respiratory illness globally. Both CD4^+^ and CD8^+^ Trm cells develop following acute influenza infection and protect against heterotypic challenge (13–16). Dysregulated CD8^+^ Trm cell responses mediate chronic inflammation and fibrotic sequelae following influenza infection in mice (19, 20). During influenza infection, Treg cells suppress effector T cell cytokine production, reduce tissue damage, promote tissue repair, and promote the resolution of inflammation following viral clearance (25–27). However, the tissue resident Treg cell response has not been characterized during influenza infection.

In this study, we utilized intravascular labeling to discriminate circulating (Circ) T cells in the lung vasculature from tissue resident (TR) T cells embedded in the lung parenchyma in conjunction with a Foxp3^RFP^/IL-10^GFP^ dual reporter mouse model in order to characterize the lung tissue resident Treg cell response during primary and secondary influenza infections (28). We found that lung tissue resident Treg cells expanded substantially post influenza infection, at a more significant rate as compared to the circulating counterpart. Moreover, the lung tissue resident Treg cells, along with tissue resident conventional T cells, are the major producers of IL-10, during the acute phases of primary and secondary influenza infections, while tissue resident Treg cell-derived IL-10 production sustained a much longer time post infection, compared to IL-10 production by conventional T cell subsets. Lung tissue resident Treg cells express high levels of immune co-inhibitory and co-stimulatory molecules including PD-1, TIGIT, LAG3, ICOS, GITR and others. Blocking PD-1 or ICOS signaling significantly altered lung tissue resident Treg cell expansion, especially the IL-10-producing population, and phenotype during secondary influenza infection, suggesting that PD-1 and ICOS signaling pathways may counter-regulate tissue resident Treg cell development and function during influenza re-infection. Utilizing MHC Class II tetramers, we also determined the influenza-specific lung tissue resident Treg and CD4^+^ conventional cell populations displayed distinct kinetics in response to secondary infection. The data presented in this report adds to our knowledge the kinetics and characteristics of lung tissue resident Treg cells during influenza infections.

## Materials and methods

### Mice

All mice were on a C57BL/6 background. Age matched male and female mice were 6-15 weeks at the time of analysis. IL-10^GFP^ (B6(Cg)-Il10tm1.1Karp/J; 014530) (29) and Foxp3^RFP^ (C57BL/6Foxp3tm1Flv/J; 008374) (30) reporter mice were purchased from the Jackson Laboratory (Bar Harbor, ME) and crossed to generate the Foxp3^RFP^/IL-10^GFP^ dual reporter strain as we previously described (31). All experiments were approved by the Institutional Animal Care and Use Committee at Louisiana State University.

### Influenza infections

All influenza infections were performed intranasally under light sedation with isoflurane as described previously (31). Mouse adapted influenza A/WSN/1933(H1N1) (WSN) and influenza A/X-31(H3N2) (X31) viral stocks were originally from the Influenza Center of Excellence & WHO Collaborating Center at St. Jude Children’s Research Hospital, and were kind gifts from Dr. David Topham at University of Rochester Medical Center and Dr. Gary Whittaker at Cornell University respectively. Influenza viruses were propagated in allantoic fluids of 10-day-old embryonated chicken eggs at 37 °C for 3 days, and plaque forming units (PFU) were quantified in MDBK cells as previously described (32). For acute infections, mice were infected with 1e3 PFU of WSN and analyzed day 7 post infection. For re-infections with heterosubtypic influenza A viruses, mice were infected with 2e2 PFU WSN (H1N1) and rested for 6 weeks, followed by re-infection 2e4 PFU of X31 (H3N2) and analyzed at the indicated timepoints post-secondary infection. Mouse weight and survival were monitored daily, and mice losing more than 30% of the original weight before the designed experimental endpoints were humanely euthanized and recorded as death incidences.

### Intravascular labeling

To stain circulating T cells *via in vivo* fluorescent staining, mice were lightly anesthetized with isoflurane five minutes prior to sacrifice, and immediately injected *via* retroorbital intravenous injection (IV) with 1.5 μg allophycocyanin (APC)-conjugated anti-CD45.2 antibody in 200 μl sterile PBS as preciously described (33).

### ICOSL and PD-L1 blockade

Mice were injected intravenously *via* retroorbital injection with the indicated doses at the indicated timepoints (see details in experimental flow illustrations) of InVivoMAb anti-mouse ICOSL (CD275) (BioXCell; clone HK5.3) or InVivoMAb anti-mouse PD-L1 (B7-H1) (BioXCell; clone 10F.9G2) in 200 μl sterile PBS. Rat IgG2a isotypes were purchased from BioXCell as recommended by the product data sheets and used as controls. Treatment antibodies (or isotypes) were given to mice at 500 μg at the first timepoint, and 250 μg at the subsequent timepoints.

### Organ collection and tissue processing

Cells from various organs were isolated as we recently described (31). Lungs, mediastinal lymph nodes, and spleens were homogenized *via* grinding against strainers, followed by filtering through the strainers. Red blood cells were lysed (RBC lysis buffer from Tonbo) before analysis. Cellular portion was resuspended in full RPMI media for staining and analysis *via* flow cytometry.

### Antibodies and reagents for flow cytometry

All fluorescent antibodies are listed in “fluorochrome-target (clone; annotation if desirable)” format below. Pacific Blue (PB)-CD103 (2E7), PerCP/Cy5.5-CD11a (M17/4), PerCP/Cy5.5-CD25 (3C7), PE-CD39 (DuHa59), Alexa Fluor (AF) 700-CD4 (GK1.5), PE-Cy7-CD69 (H12F3), BV421-CXCR6 (SA051D1), PE-Cy7-GITR (DTA-1), BV421-ICOS (C398.4A), PE-Cy7-LAG-3 (C9B7W), APC-Cy7-PD-1 (29F.1A12), APC-Cy7-TCRβ (H57-597) and APC-CD45.2 (104) were from BioLegend. PerCP/Cy5.5-T-bet (O4-46) was from BD Pharmingen. violetFluor (vF) 450-CD25 (PC61.5), PE-CD25 (PC61.5), APC-Cy7-CD4 (GK1.5), FITC-CD4 (RM4-5), PerCP/Cy5.5-CD8α (53-6.7), APC-Cy7-CD8α (53-6.7), PE-CTLA-4 (UC10-4F10-11), and APC-TIGIT (1G9) were purchased from Tonbo Biosciences. PE-eFluor610-Foxp3 (FJK-16s) and AF700-Ki-67 (SolA15) were from ThermoFisher Scientific.

*Other reagents*: Ghost Dye™ Violet 510 viability dye was from Tonbo Biosciences. The following reagent(s) was obtained through the NIH Tetramer Core Facility: PE-labeled I-A(b) Influenza A nucleocapsid (NP) 311-325 QVYSLIRPNENPAHK loaded class II tetramer for NP-specific CD4^+^ T cell detection.

### Flow cytometry

Surface staining of live cells were done in the presence of Fc block and fixable viability dye. For intracellular cytokine staining, cells were stimulated with cell stimulation cocktail (Tonbo Biosciences) for 4-5 hours, subjected to surface marker staining in the presence of Fc block, fixed in 4.2% paraformaldehyde, permeabilized and stained with the indicated anti-cytokine antibodies in intracellular perm/wash buffer (BioLegend) for 2 hours at 4 °C. Nuclear staining was performed with Foxp3 staining buffer kit (eBioscience). T cells were identified *via* lymphocyte/single cells/live cells/TCRβ^+^ gating. CD4^+^Foxp3^RFP+^ T cells were defined as Foxp3^+^ gating Treg cells, while CD4^+^Foxp3^RFP-^ T cells were defined as conventional CD4^+^ T cells. Tissue resident or circulating status was then determined *via* fluorescent *in vivo* IV staining of CD45.2 (CD45.2^IV-^ for tissue resident cells and CD45.2^IV+^ for circulating cells respectively). IL-10^GFP^ was utilized to analyze *ex vivo* IL-10 production by T cells during influenza infections.

### Histology

Left lung lobes were collected and fixed in 10% formalin. Lung sections were stained with hematoxylin and eosin (H&E) and scored by a board-certified pathologist at the Louisiana Animal Disease Diagnostic Laboratory, in a blinded manner. The scoring interactively evaluates the histopathologic levels of bronchiolar necrosis, alveolar bronchiolization/squamous metaplasia, peribronchiolar/perivascular cuffing, and alveolar inflammation. Scores were added together to achieve a cumulative score, at the range of 0 to 24.

### Statistical analysis

T-test, one-way ANOVA and two-way ANOVA with Tukey’s *post hoc* test were performed with GraphPad Prism 8 with *p* ≤ 0.05 considered statistically significant. “NS” refers to “No Significance”.

## Results

### Lung tissue resident Treg cells rapidly expand during acute primary influenza infection

**Figure 1:**
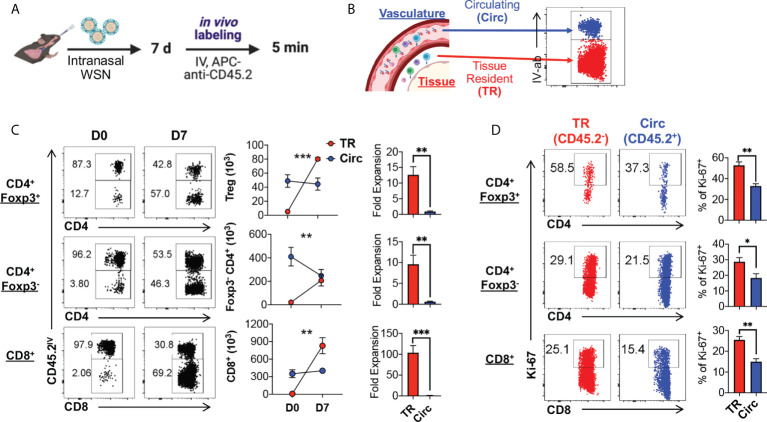
Lung tissue resident Treg cells rapidly expand upon primary acute influenza infection. **(A)** Schematics of primary infection and *in vivo* labeling of circulating immune cells: Foxp3^RFP^/IL-10^GFP^ dual reporter mice were infected with 10^3^ PFU of the mouse-adapted WSN (H1N1) virus intranasally (IN), and 7 days post infection, mice were injected with an APC-anti-CD45.2 antibody intravenously (IV), followed by euthanasia 5 minutes after the antibody injection. **(B)** Illustration of lung immune cell staining for cells residing in the vasculature versus tissue. Circulating (Circ) cells will be exposed to the intravenous antibody staining, while the tissue resident (TR) cells were blocked from staining, resulting in labeling of the circulating cells only. After gating on live singlet T cells, a representative plot of the Circ and TR T cells extracted from the lungs of the infected mice is shown. **(C)** Representative flow plots and summary of numbers and fold expansion of Circ versus TR CD4^+^ Foxp3^+^, CD4^+^ Foxp3^-^, and CD8^+^ T cells isolated from the lungs of Foxp3^RFP^/IL-10^GFP^ dual reporter mice prior to (D0) and 7 days post (D7) acute flu infection. The average number of cells on D0 was set as “1” for fold change calculation. **(D)** Representative flow plots and summary of Ki-67 expression by Circ and TR cell subsets as in **(C)**. N ≥ 3. Data represent results of more than three experiments. *p ≤ 0.05, **p ≤ 0.01, ***p ≤ 0.001, by two-way ANOVA or unpaired t test. Data presented as Mean ± S.E.M.

The kinetics of tissue resident Foxp3-expressing Treg (TR-Treg) cells in the lungs during acute primary influenza infection is not as well characterized as the tissue resident memory CD8^+^ T cells. To determine whether and to what scale TR-Treg cells expand in the lungs of mouse model of primary acute influenza infections, we used Foxp3^RFP^/IL-10^GFP^ dual reporter mice, infected them intranasally (IN) with the mouse-adapted WSN (H1N1) strain of influenza A and analyzed 7 days post infection (dpi) with *in vivo* fluorescent anti-CD45.2 antibody staining *via* intravenous (IV) injection to label the circulating T cells in the lungs (**Figure 1A**). With IV anti-CD45.2 antibody treatment, immune cells circulating in the pulmonary vascular system will be accessed by the fluorescent antibody and stained positive, while lung tissue resident cells are inaccessible and will be labeled negative (**Figure 1B**). With this protocol, we found that the number of circulating (Circ) Foxp3^+^ Treg cells and CD8^+^ cells remained stable during acute infection compared naïve mice, while their lung tissue resident (TR) counterparts expanded ~12.63 and ~103.85 folds, respectively (**Figure 1C**). Notably, the TR-Treg cells are significantly expanded following acute primary influenza infection, although not as prominent as the TR-CD8^+^ T cells (**Figure 1C**). Using nuclear staining for Ki67, we also observed a significantly higher rate of T cell expansion in the tissue residents than their circulating counterparts, including the CD4^+^ Foxp3^+^ Treg, CD4^+^ conventional T, and the CD8^+^ T cell subsets (**Figure 1D**).

**Figure 2:**
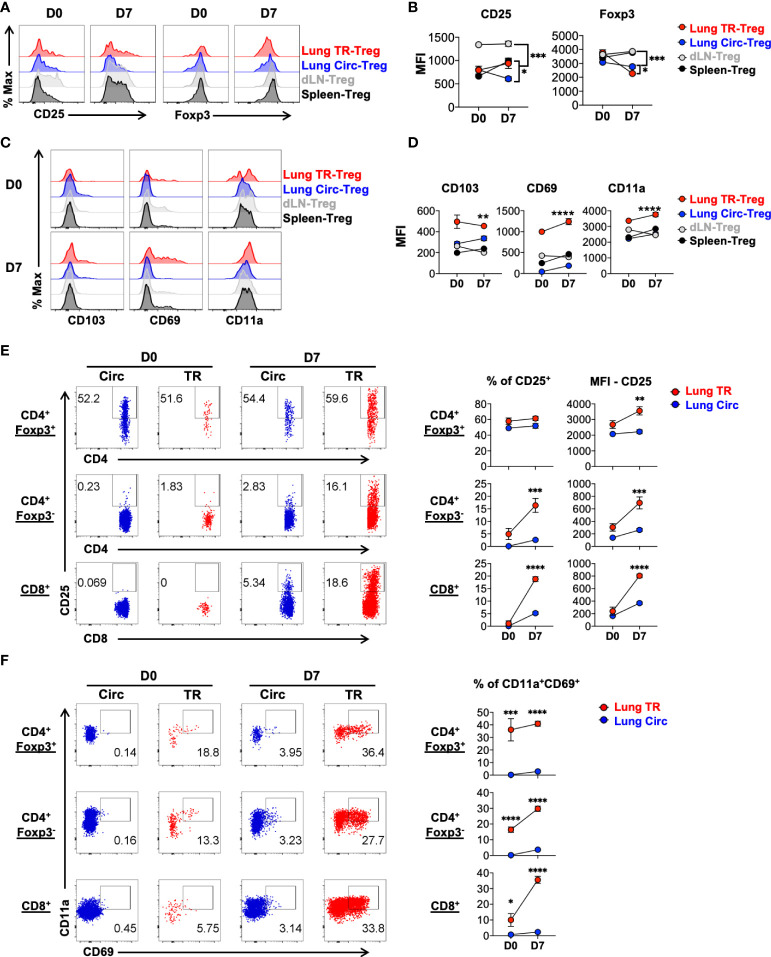
Characterization of signature marker expression by lung tissue resident Treg cells during acute primary influenza infection. Mice were infected and treated as in **Figure 1**, and cells from lung, draining lymph node (dLN) and spleen were isolated from mice prior to (D0) and 7 days post (D7) acute flu infection. Live singlet T cells were gated for analyses. **(A, B)** Representative flow histograms and summary of levels of expression of Treg signature markers, CD25 and Foxp3 (MFI: mean fluorescence intensity), by lung TR-Treg, lung Circ-Treg, dLN-Treg, and spleen-Treg cells. **(C, D)** Representative flow histograms and summary of levels of expression of tissue resident T cell signature markers, CD103, CD69 and CD11a (MFI), by the Treg subsets as described in **(A, B)**. **(E, F)** Representative flow plots of CD25, CD69 and CD11a expression by lung Circ and TR CD4^+^ Foxp3^+^, CD4^+^ Foxp3^-^, and CD8^+^ T cells. N ≥ 3. Data represent results of more than three experiments. *p ≤ 0.05, **p ≤ 0.01, ***p ≤ 0.001, ****p ≤ 0.0001, by two-way ANOVA. Data presented as Mean ± S.E.M.

To further compare the levels of Treg cell and tissue resident T cell featured markers in the lung TR-Treg cell subset to other Treg cell subsets, including the lung circulating Treg cells and Treg cells found in the lung draining lymph nodes (dLN) and and spleen of the same animals, we gated on the Foxp3^RFP+^ CD4^+^ T cells in these compartments and analyzed their levels of expression of CD25, Foxp3, CD103, CD69 and CD11a. The lung TR-Treg cells expressed higher levels of the high affinity IL-2 receptor, CD25, compared to their circulating counterpart in the lung of mice with acute influenza infection, but this level of CD25 expression in lung TR-Treg is comparable to that observed in splenic Treg cells (**Figure 2A, B**). Interestingly, during acute influenza infection, lung Treg cells, including the TR-Treg and Circ-Treg cell subsets displayed decreased Foxp3 expression while Foxp3 expression remained stable in Treg cells extracted from the lymph nodes and spleens (**Figure 2A, B**), which is however consistent with previous findings that Foxp3 expression is downregulated during inflammation (34, 35). In contrast, the levels of expression of CD103, CD69 and CD11a were higher in the lung TR-Treg cells, as compared to their circulating counterpart in the lung or Treg cell subsets in the dLN and spleens during acute infection (**Figure 2C, D**). While compared to the lung tissue resident conventional T cells counterparts (**Figure 2E**, middle row), lung tissue resident Treg cells (**Figure 2E**, top row)exhibited higher frequency and level of CD25 expression, but not notably higher levels of conventional tissue resident markers CD69/CD11a (**Figure 2F**). Together, these data indicate that TR-Treg cells expand and express surface markers classically associated with tissue residency during primary acute flu infection.

### Lung tissue resident Treg cells express high levels of IL-10, ICOS, and PD-1 during acute influenza infection

**Figure 3:**
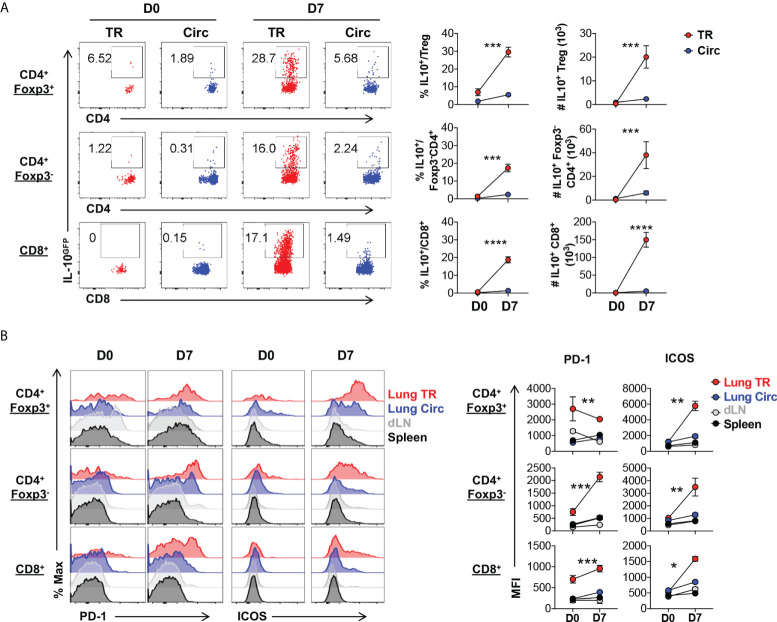
Lung tissue resident Treg cells display an effector phenotype with high levels of IL-10, PD-1 and ICOS expression. T cells from **Figure 2** were further analyzed. **(A)** Representative flow plots and summary of IL-10^GFP^ expression and numbers of IL-10^+^ lung Circ and TR CD4^+^ Foxp3^+^, CD4^+^ Foxp3^-^, and CD8^+^ T cells. **(B)** Representative flow histograms and summary of PD-1 and ICOS expression (MFI) by lung tissue resident (TR), lung circulating (Circ), dLN, and spleen CD4^+^ Foxp3^+^, CD4^+^ Foxp3^-^, and CD8^+^ T cells. N ≥ 3. Data represent results of more than three experiments. *p ≤ 0.05, **p ≤ 0.01, ***p ≤ 0.001, ****p ≤ 0.0001, by two-way ANOVA. Data presented as Mean ± S.E.M.

During acute viral inflammation, Treg cells acquire an effector phenotype characterized by upregulation of the immunomodulatory effector cytokine IL-10, along with costimulatory and co-inhibitory molecules (36–39). Using the Foxp3^RFP^/IL-10^GFP^ dual reporter mouse model, we tracked the levels of IL-10 production in Treg cells during acute influenza infection and found that the lung tissue resident Foxp3^+^ Treg cells, along with the conventional CD4^+^ and CD8^+^ tissue resident T cells are the major producers of IL-10 during acute flu in mice (**Figure 3A**). The lung tissue residents produced significantly higher levels of IL-10 than their circulating counterparts (**Figure 3A**). Previous work has found most tissue resident Treg cells display an effector-like phenotype even at steady state, as reflected by the high levels of expression of Treg cell functional feature markers such as the immune checkpoint proteins (4, 36, 40). Co-stimulatory molecules such as ICOS and the TNFRSF family member GITR are highly expressed by tissue resident Treg cells of mice and humans, and play an important role in their maintenance in NLT (38, 39, 41–50). In agreement with these, compared to circulating Treg cells, we found that lung tissue resident Treg cells express higher levels of PD-1, TIGIT and GITR, even at the naïve state, while ICOS and LAG-3 are significantly upregulated following acute influenza infection (**Supplemental Figure 1**). Notably, lung tissue resident Treg cells maintain a high level of PD-1 expression regardless of flu infection, but significantly upregulate the level of expression of ICOS significantly upon acute flu (**Figure 3B**). In contrast, the lung tissue resident conventional T cells upregulated the expression of both PD-1 and ICOS to a moderate level upon acute flu, with the exception of tissue resident conventional CD4^+^ T cells which expressed comparable levels of PD-1 to TR-Treg cells (**Figure 3B**). These results show that TR-Treg cells display an effector-like phenotype during steady state, and acquire more effector associated features during acute primary influenza infection.

### Lung tissue resident Treg cells accumulate following resolution of acute influenza

**Figure 4:**
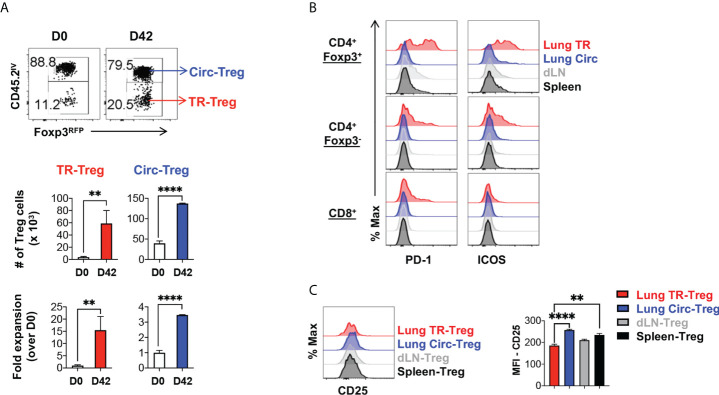
Lung tissue resident Treg cells do not contract and maintain high levels of PD-1 and ICOS expression, following resolution of acute influenza. Foxp3^RFP^/IL-10^GFP^ dual reporter mice were infected with 200 PFU of the mouse-adapted WSN virus (IN), and 42 days post infection, mice were injected with an APC-anti-CD45.2 antibody intravenously (IV), followed by euthanasia 5 minutes after the antibody injection. **(A)** Representative flow plots and summary of numbers and fold expansion of lung Circ versus TR Treg cells prior to (D0) infection and after resolution of acute influenza (D42). The average number of cells on D0 was set as “1” for fold change calculation. **(B)** Representative flow histograms of PD-1 and ICOS expression by lung tissue resident (TR), lung circulating (Circ), dLN, and spleen CD4^+^ Foxp3^+^, CD4^+^ Foxp3^-^, and CD8^+^ T cells. **(C)** Representative flow histograms and summary of CD25 expression (MFI) by lung TR-Treg, lung Circ-Treg, dLN-Treg, and spleen-Treg cells. N ≥ 3. Data represent results of three experiments. **p ≤ 0.01, ****p ≤ 0.0001, by unpaired student *t* test. Data presented as Mean ± S.E.M.

Following resolution of inflammation, it has been suggested that tissue resident Treg cells population contracts and downregulates effector molecules (4, 51). A stepwise model has been proposed where local inflammatory and tissue environment signals promote the differentiation of tissue resident Treg cells (40, 52, 53). However, it was unclear whether lung tissue resident Treg cells following acute flu and resolution of the flu-associated lung inflammation would follow a similar kinetics of contraction. When we tracked the Foxp3^RFP+^ Treg cells in the flu-infected and recovered animals, we found that 42 days post primary infection, there was a significantly increased number of both tissue resident and circulating Treg cells in the lung of the infected animals (**Figure 4A**), compared to naïve mice. Notably, the lung tissue resident Treg subset maintained at high levels of fold expansion (~ 15-fold) compared to that observed in naïve animals (**Figure 4A**). This level is similar to the fold expansion 7 days post primary influenza infection (**Figure 1C**), suggesting that, following acute flu, lung tissue resident Treg cells do not undergo substantial contraction following the resolution of flu infection and the associated inflammation. Moreover, these lung tissue resident Treg cells maintained high levels of expression of both PD-1 and ICOS (**Figure 4B**). In contrast, the expression of CD25 was lower on lung tissue resident Treg cells compared to their circulating and splenic counterparts (**Figure 4C**) suggesting that IL-2 signaling may not be the predominant signal for lung tissue resident Treg cell maintenance following influenza infection (39).

Given the prominent levels of PD-1 and ICOS expression in the lung tissue resident Treg cells, following acute influenza infection, we sought to determine whether blockade of PD-1 or ICOS signaling would have significant effects on lung tissue resident Treg cell development and function during this stage. PD-L1 blockade was performed *via* administration of αPD-L1 antibodies during primary infection (**Supplemental Figure 2A**). There was no significant difference in the number of lung TR versus Circ Treg cells, despite a slight increase by αPD-L1, between the IgG isotype control and αPD-L1 treated groups (**Supplemental Figure 2B**). No difference in frequency or number of IL-10 producing TR-Treg cells were observed either, between the isotype and αPD-L1 treated groups (**Supplemental Figure 2C**). Similarly, to probe the role of ICOS signaling, αICOSL was administered during primary influenza infection (**Supplemental Figure 2D**). There was a trend towards a reduced number of both lung TR and Circ Treg cells (**Supplemental Figure 2E**), which is not statistically significant either. Again, no significant difference in the frequency or number of IL-10 producing TR-Treg cells was observed (**Supplemental Figure 2F**). These data suggest that, during primary acute influenza infection, despite the high levels of PD-1 and ICOS expression, PD-1 and ICOS signaling pathways do not have prominent function in regulating lung tissue resident Treg cells development. However, at this point, it is unclear whether they play more significant roles during the later stage following resolution of the acute infection and upon secondary infections.

### Lung tissue resident Treg cells rapidly expand and contract upon secondary influenza infection

**Figure 5:**
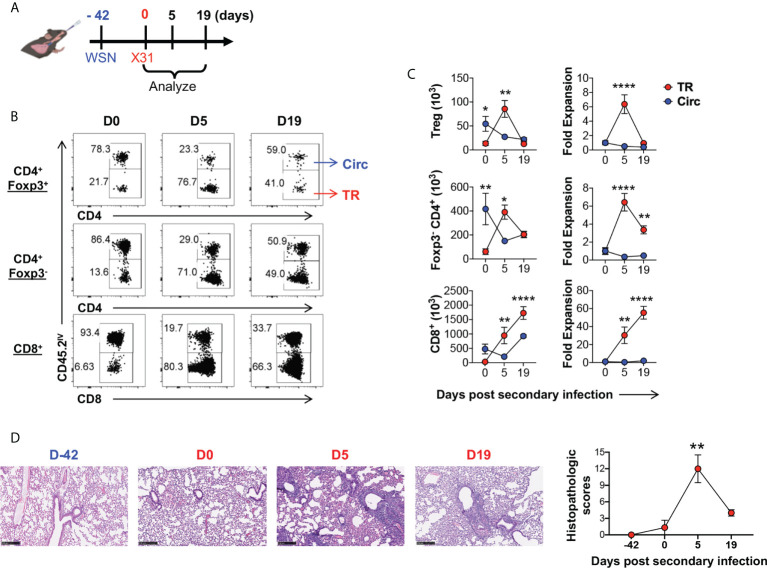
Lung tissue resident Treg cells rapidly expand and contract following secondary influenza infection. **(A)** Schematics of heterologous secondary influenza infection and analyses: Foxp3^RFP^/IL-10^GFP^ dual reporter mice were infected with 200 PFU of the mouse-adapted WSN virus (H1N1, IN), and 42 days later, were further infected with 2 × 10^4^ PFU of the mouse-adapted X31 virus (H3N2, IN). Mice were analyzed prior to (D0), and 5 and 19 days post the secondary infection. **(B)** Representative flow plots of *in vivo* staining of CD45.2 in CD4^+^ Foxp3^+^, CD4^+^ Foxp3^-^, and CD8^+^ T cells isolated from the lungs of Foxp3^RFP^/IL-10^GFP^ dual reporter mice prior to (D0) and 5 (D5) and 19 (D19) days post secondary flu infection. **(C)** Numbers and fold expansion of Circ versus TR CD4^+^ Foxp3^+^ (Treg), CD4^+^ Foxp3^-^, and CD8^+^ T cells isolated from the lungs of Foxp3^RFP^/IL-10^GFP^ dual reporter mice at the indicated time points following secondary flu infection. The average number of cells on D0 was set as “1” for fold change calculation. N ≥ 3. Data represent results of three experiments. *p ≤ 0.05, **p ≤ 0.01, ****p ≤ 0.0001, by two-way ANOVA with *post-hoc* tests for time point-pairwise comparisons. Data presented as Mean ± S.E.M. **(D)** Representative histological images (H&E staining) and accumulative histopathologic scores of the lungs collected from mice at the indicated time points. Scale bar = 250 μm. N ≥ 3. Data represent results of two experiments. **p ≤ 0.01, by one-way ANOVA, compared to baseline levels (Day -42). Data were presented as Mean ± S.E.M.

Unlike tissue resident memory T cell response, the tissue resident Treg cell response has not been characterized during secondary infection (14–16, 54), therefore, we proceeded to characterize the kinetics of lung tissue resident Treg cell development during secondary influenza infection. To circumvent humoral immune response that would diminish homologous secondary infections, we utilized a heterologous infection model in which mouse-adapted WSN (H1N1) was used in primary infection, followed by X31 (H3N2) secondary infection 6 weeks post the primary infection (**Figure 5A**). We found that lung tissue Treg cells rapidly expanded upon secondary infection (5 days post infection), at a significantly higher rate as compared to their circulating counterpart in the lungs of the same animals, and this expansion was followed by a population contraction (19 days post infection) (**Figure 5B, C**). This kinetics is similar to the lung tissue resident conventional CD4^+^ T cell subset in the lungs of the same infected animals (similar fold expansion at ~6.36 and ~6.42 fold respectively). In contrast, their circulating counterparts contracted modestly following secondary influenza infection (**Figure 5C**). Interestingly, the numbers of lung tissue resident CD4^+^ T cells, including the Treg and conventional cell subsets, peaked earlier upon secondary infection than lung tissue resident CD8^+^ T cells and returned towards baseline numbers by day 19, while lung tissue resident CD8^+^ T cells remained elevated (**Figure 5C**). Histological examination revealed that, by the time of the secondary infection, lung inflammation had been completely resolved; and following the secondary infection, lung inflammation peaked at day 5 post infection, and had largely resolved by day 19 mirroring the kinetics of the TR-Treg cell expansion (**Figure 5D**). These data suggested that lung tissue resident Treg cells are one of the first T cell responders to secondary influenza infection and may play a role in dictating immune regulation in response to secondary flu and the associated immunopathology.

One of the major questions is whether the lung tissue resident Treg cells function in an antigen-specific manner. Other groups have described influenza antigen-specific Treg cells during secondary infection (55), however the lung tissue residency status was not explored. We were able to detect influenza A NP_311-325_ epitope-specific Treg and conventional CD4^+^ T cells in the lung tissue, vascular, and SLO (**Supplemental Figure 3**). Interestingly, the majority of these NP-specific CD4^+^ T cells are lung tissue residents peaking during the effector immune stage following secondary flu infection, in both the Treg and conventional CD4^+^ T cell subsets. At five days post secondary infection, Treg and CD4^+^ conventional T (Tcon) cells that were lung tissue residents exhibited the highest percentages of NP_311-325_ tetramer bound fractions (**Supplemental Figure 3B**, left column), although, by number, there were more NP_311-325_ -specific CD4^+^ Tcon cells observed in the spleen (**Supplemental Figure 3B**, middle column); at this time, NP_311-325_ -specific lung tissue resident Treg cells had expanded ~6 fold (**Supplemental Figure 3B**, right column) similar to the lung tissue resident Treg population as a whole (**Figure 5C**). However, this was less than the NP_311-325_ -specific lung tissue resident conventional CD4^+^ T cells which expanded ~19 fold at this time point (**Supplemental Figure 3B**, right column). By 19 days post secondary flu infection, NP_311-325_ -specific lung tissue resident Treg cells had contracted to the level comparable to that observed prior to the re-infection, while NP_311-325_ -specific lung tissue resident conventional CD4^+^ T cells persisted. Our results indicate that influenza specific lung tissue resident Treg and conventional CD4^+^ T cell populations display distinct kinetics in response to secondary influenza infection. The fact that influenza antigen-specific lung tissue resident Treg cells expanded and contracted following the footprints of active flu infection, suggest that their expansion may be regulated in an antigen-specific manner, and in return, they may play a role in antigen-specific immunomodulation of the lung immune responses to flu re-infection.

### Lung tissue resident Treg cells express high levels of IL-10, PD-1 and ICOS during secondary influenza infection

**Figure 6:**
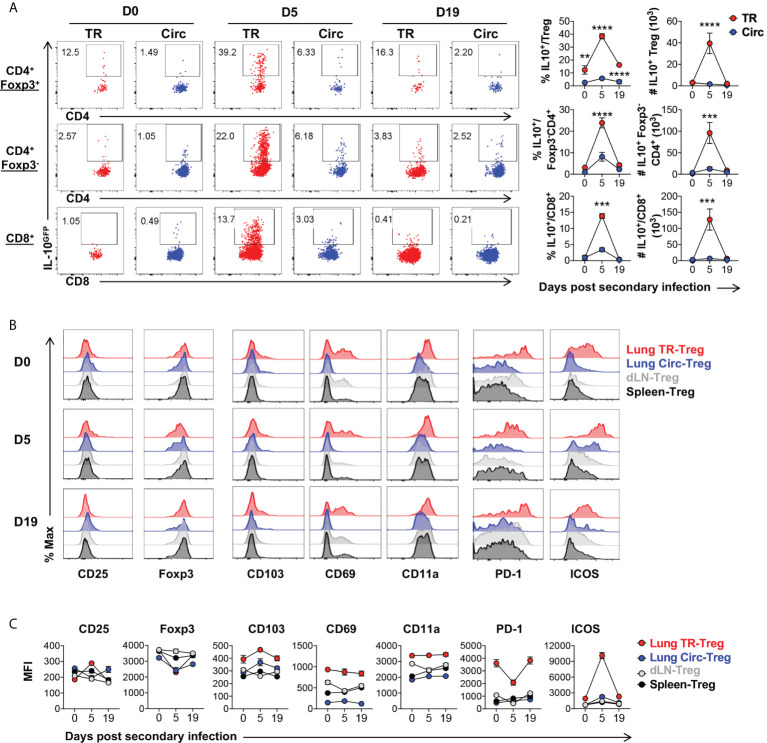
Characterization of lung tissue resident Treg cells following secondary influenza infection. T cells from **Figure 5** were further analyzed. **(A)** Representative flow plots and summary of IL-10^GFP^ expression and numbers of IL-10^+^ lung Circ and TR CD4^+^ Foxp3^+^, CD4^+^ Foxp3^-^, and CD8^+^ T cells, on D0, D5 and D19 during influenza secondary infection. **(B, C)** Representative flow histograms and summary of MFI of CD25, Foxp3, CD103, CD69, CD11a, PD-1 and ICOS expression by lung TR-Treg, lung Circ-Treg, dLN-Treg, and spleen-Treg cells. N ≥ 3. Data represent results of three experiments. **p ≤ 0.01, ***p ≤ 0.001, ****p ≤ 0.0001, by two-way ANOVA with *post-hoc* tests for time point-pairwise comparisons. Data presented as Mean ± S.E.M.

Similar to the case in primary infection, lung tissue resident T cells, including Treg, CD4^+^Foxp3^-^, and CD8^+^ T cell subsets all expanded following secondary influenza infection. These lung tissue resident T cells also significantly upregulated IL-10 expression, compared to their circulating counterparts isolated from the lungs of the same animals (**Figure 6A**). Among the lung tissue resident T cell subsets, the tissue resident Treg cells displayed the highest frequency of IL-10-producing cells (**Figure 6A**). Five days post secondary infection, CD25 were slightly upregulated while Foxp3 was downregulated in the lung tissue resident Treg cells, which are both adjusted back to a level comparable to that observed right before the secondary infection, by 19 days post infection (**Figure 6B, C**). Expression of tissue resident T cell markers CD103, CD69 and CD11a remained stable at high levels in the lung tissue resident Treg cells during secondary infection, as compared to the lung circulating Treg, dLN-Treg and spleen-Treg cell subsets (**Figure 6B, C**, column 3-5). More strikingly, PD-1 and ICOS remained at significantly high levels in lung tissue resident Treg cells, as compared to other T cell subsets (**Figure 6B, C**, last two columns); PD-1 was rapidly downregulated while ICOS was up-regulated by 5 days post secondary infection, and adjusted back to the levels observed before re-infection by 19 days post secondary infection (**Figure 6B, C**) which are counter-correlated to the kinetics of lung tissue resident Treg cell expansion and IL-10 expression following secondary flu. These data suggest that lung tissue resident Treg cells may play a critical role in modulating the immune responses to flu re-infections *via* IL-10 production, and their development and function may be counter-regulated by PD-1 and ICOS signaling during flu re-infections.

### PD-1 and ICOS signaling counter-regulates lung tissue resident Treg cell survival and IL-10 production

**Figure 7:**
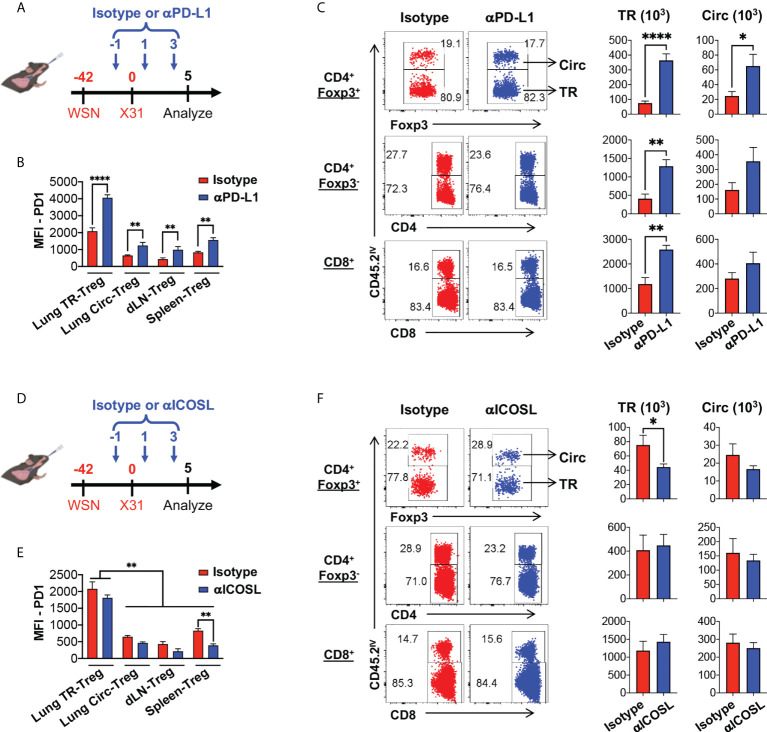
PD-1 and ICOS signaling counter-regulates lung tissue resident Treg cell expansion and IL-10 production during secondary influenza. **(A–C)** PD-1 signaling blockade enhances lung tissue resident Treg cells expansion during secondary influenza infection. **(A)** Schematics of PD-1 signaling blockade during secondary influenza infection: Foxp3^RFP^/IL-10^GFP^ dual reporter mice were infected with 200 PFU of WSN virus (H1N1, IN), and 42 days later, were further infected with 2 × 10^4^ PFU of X31 virus (H3N2, IN). The date of secondary infection was set as “D0”. Anti-PD-L1 antibody (or isotype control) was injected intravenously on D-1, D1 and D3, while mice were analyzed on D5. **(B)** Summary of PD-1 expression (MFI) by lung TR-Treg, Circ-Treg, dLN-Treg, and spleen-Treg cells, isolated from mice that received either the isotype or anti-PD-L1 antibody treatment. **(C)** Representative flow plots of *in vivo* staining of CD45.2 and summary of numbers of TR versus Circ CD4^+^ Foxp3^+^ (Treg), CD4^+^ Foxp3^-^, and CD8^+^ T cells isolated from the lungs of the isotype or anti-PD-L1 antibody-treated mice. **(D–F)** ICOS signaling blockade attenuates lung tissue resident Treg cells expansion during secondary influenza infection. **(D)** Schematics of ICOS signaling blockade during secondary influenza infection: Foxp3^RFP^/IL-10^GFP^ dual reporter mice were infected as indicated in **(A)**. The date of secondary infection was set as “D0”. Anti-ICOSL antibody (or isotype control) was injected intravenously on D-1, D1 and D3, while mice were analyzed on D5. **(E)** Summary of PD-1 expression (MFI) by lung TR-Treg, Circ-Treg, dLN-Treg, and spleen-Treg cells, isolated from mice that received either the isotype or anti-ICOSL antibody treatment. **(F)** Representative flow plots of *in vivo* staining of CD45.2, and summary of numbers of TR versus Circ CD4^+^ Foxp3^+^ (Treg), CD4^+^ Foxp3^-^, and CD8^+^ T cells isolated from the lungs of the isotype or anti-ICOSL antibody-treated mice. N ≥ 3. Data were combined from three different experiments. *p ≤ 0.05, **p ≤ 0.01, ****p ≤ 0.0001, by unpaired student *t* test. Data presented as Mean ± S.E.M.

**Figure 8:**
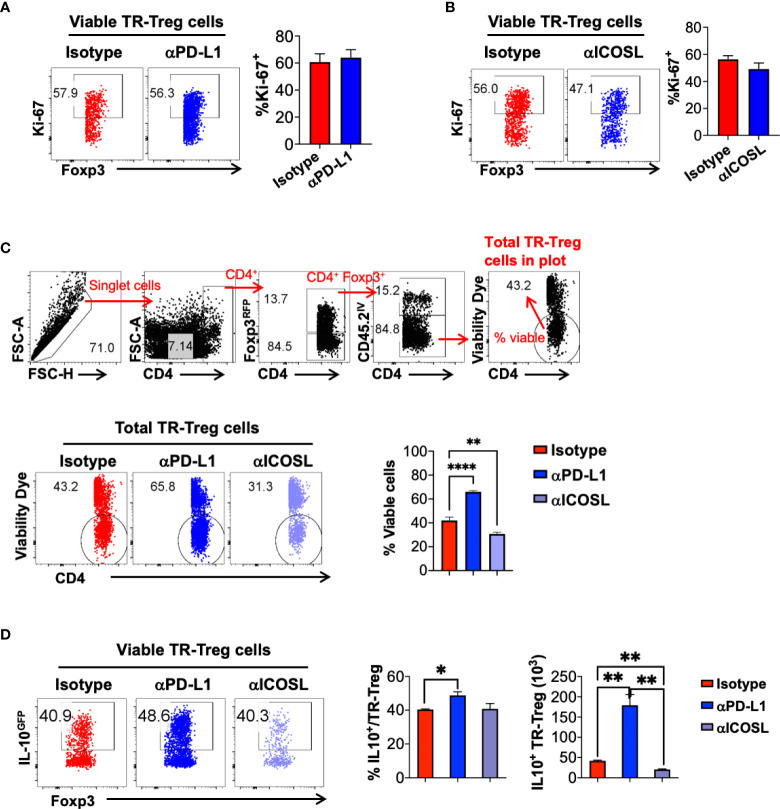
PD-1 and ICOS signaling pathways counter-regulate tissue resident Treg cell viability and IL-10 production during secondary influenza infection. Mice were treated with isotype, anti-PD-L1 or anti-ICOSL during secondary influenza infection, as shown in **Figure 7**. **(A, B)** Representative flow plots and summary of frequency of Ki-67^+^ lung tissue resident Treg cells in mice treated with Isotype, **(A)** anti-PD-L1 or **(B)** anti-ICOSL antibody. N ≥ 3. Data represent the results of at least two different experiments and are presented as Mean ± S.E.M. **(C)** Gating strategy to determine lung tissue resident Treg cell viability, representative flow plots and summary of frequency of viable lung tissue resident Treg cells in mice treated with the indicated antibodies. **(D)** Representative flow plots of IL-10^GFP^ expression by lung TR-Treg cells, in mice receiving isotype, anti-PD-L1 or anti-ICOSL treatment during secondary influenza infection. Summary of the percentage of IL-10^+^ fraction and numbers of IL-10^+^ lung TR-Treg cells under the indicated treatment conditions were shown as well. N ≥ 3. Data were combined from three different experiments. *p ≤ 0.05, **p ≤ 0.01, ****p ≤ 0.0001, by unpaired student *t* test. Data presented as Mean ± S.E.M.

To further define the role of PD-1 and ICOS signaling pathways in lung tissue resident T cell expansion and IL-10 production during flu re-infection, we tested the effects of PD-1 and ICOS signaling blockades during secondary influenza infection (**Figure 7A, D** respectively). Administration of neither αPD-L1 nor αICOSL significantly affected weight loss during secondary infection (**Supplementary Figure 4A, D**). When αPD-L1 was administered, the levels of PD-1 expression by Treg cells (**Figure 7B**; **Supplemental Figure 4B**) and the total numbers (**Figure 7C**) of lung tissue resident Treg, CD4^+^ Foxp3^-^, and CD8^+^ T cells significantly increased, as compared to when the isotype antibody was administered (**Figure 7B, C**). In contrast, blocking ICOS signaling by αICOSL antibody did not affect PD-1 expression as drastically (**Figure 7E**; **Supplemental Figure 4E**), but resulted in a significantly impaired expansion of the lung tissue resident Treg cell population (**Figure 7F**). Interestingly, neither αPD-L1 nor αICOSL antibody treatment affected ICOS expression on the tissue resident Treg cells (**Supplemental Figure 4C, F**). We did not find a significant difference in the expression of proliferation marker Ki-67 among the viable tissue resident Treg population between isotype and αPD-L1 nor αICOSL antibody treated mice (**Figure 8A, B**), at the end point that we analyzed. It is possible that PD-1 and ICOS signaling pathways are not involved in regulating TR-Treg cell proliferation; alternatively, it is also possible that TR-Treg cell proliferation occurred earlier prior to the time point of analyses, and PD-1 and ICOS played a role in regulating lung TR-Treg cell proliferation during the earlier stage following secondary flu infection. However, when we expanded the gating to include all tissue resident Foxp3^+^ T cells and further examine the rate of viable cells (see gating strategy in **Figure 8C**), we found that PD-1 and ICOS counter-regulate the viability of the lung TR-Treg cells during secondary flu infection (**Figure 8C**).

Furthermore, we analyzed the levels of IL-10 production in the lung tissue resident Treg cells during effector stage of the secondary influenza infection, and found that blocking PD-1 signaling resulted in a modest but statistically significant increase in the frequency of IL-10 producing lung tissue resident Treg cells, while blocking ICOS signaling did not affect this parameter, as compared to the isotype antibody-treated group (**Figure 8D**). However importantly, given the significantly impacts of PD-1 and ICOS signaling blockade in the expansion of the lung tissue resident Treg cells, the absolute numbers of IL-10-producing lung tissue resident Treg cells were significantly increased in the αPD-L1-treated group while decreased in the αICOSL group, compared to the number in the isotype-treated group (**Figure 8D**). Note that PD-1 blockade has a predominant function in lung tissue resident T cells, including Treg and conventional T cell subsets, while ICOS blockade has more selective impacts specifically on the lung tissue resident Treg cells (**Figure 7C, F**). However, with αPD-L1 or αICOSL treatment, we did not not observe a significant difference in the frequency or number of IL-10-producing tissue resident conventional CD4^+^ or CD8^+^ T cells (**Supplemental Figure 5**), suggesting that the counter regulatory effects of PD-1 and ICOS signaling on IL-10 production is tissue resident Treg cell specific. Taken together, our data suggest that PD-1 and ICOS signaling pathways counter-regulate lung tissue resident Treg cell survival and function, in particular, the number of IL-10-producing lung tissue resident Treg cell population during secondary flu infection.

## Discussions

There is growing interest in the role of tissue resident Treg cells in tissue homeostasis and inflammation (56), however, our knowledge of the kinetics and characteristics of lung tissue resident Treg cells development and function during repetitive flu infections was rather limited. In this work, we characterized the lung tissue resident Treg cells response during acute primary and repeated influenza infections. We found that lung tissue resident Treg cells can rapidly expand during the effector phases of immune responses following both primary and secondary flu infections, while the lung circulating Treg cells do not expand. During primary flu infection, the lung tissue resident Treg display significantly higher levels of expression of multiple effector molecules including IL-10, PD-1 and ICOS. Following resolution of the primary flu infection, there was an accumulation of lung tissue resident Treg cells expressing high levels of PD-1 and ICOS, consistent with previous reports that ICOS^high^ Treg cells could accumulate at barrier tissues in both humans and mice (47, 48, 50). PD-1 is constantly expressed at high levels in lung tissue resident Treg cells, as compared to other lung tissue resident T cell types or Treg cells in the other compartments. During the effector phases of immune responses to primary and secondary flu infections, PD-1 expression in lung tissue resident Treg cells is slightly down-regulated, although remains at high levels of expression, which shows an inverse correlation to lung tissue resident Treg cell expansion and IL-10 production. Indeed, blocking PD-1 resulted in a trend of increased expansion of lung tissue resident Treg cells during primary flu and a significant increase in expansion and IL-10 production in lung tissue resident Treg cells during secondary flu infection. On the other hand, ICOS is upregulated during lung tissue resident Treg expansion in the effector phases of immune responses to primary and secondary flu infection and blocking ICOS signaling resulted in an attenuated expansion of lung tissue resident Treg cells, in particular, the IL-10-producing subset.

Our data suggest that PD-1 signaling plays a predominant role in negatively regulating lung tissue resident Treg cell survival and IL-10 production during flu, especially during re-infections; while ICOS signaling promotes tissue resident Treg cell survival and IL-10 production during this process. At homeostasis, Treg cell-specific deletion of PD-1 results in increased accumulation of effector-like Treg (eTreg) cells (57), while Treg cell-specific deletion of ICOS results in minor reduction of Treg cell frequency but not total number (58).

PD-1 signaling has also been shown to limit the size of the Treg cell population during both chronic and acute infections (57). PD-L1 blockade during chronic LCMV infection expands the liver and lung TR-Treg cell population, potentially through increased proliferation (59). During acute *Toxoplasma gondii* infection, the PD-1^hi^ eTreg cell population rapidly contracts and could be rescued *via* PD-L1 blockade (57). PD-L1 blockade coincides with increased proliferation and a reduction in expression of the proapoptotic molecule BIM in Treg cells (57). We observed a similar reduction in PD-1^hi^ TR-Treg cells during secondary infection (**Figure 6B, C**) as well as improved survival but did not find evidence of increased proliferation (**Figure 8A, C**). PD-1 signaling has been implicated in balancing CD8^+^ Trm mediated protection and immunopathology during secondary influenza infection (20). PD-L1 blockade expands tissue resident Treg cells during chronic viral infection (59). Our results further extended our understanding of the role of PD-1 signaling in lung tissue resident Treg cell expansion and function during flu infections.

The role of ICOS signaling in Treg cells differs between subsets and tissue environment. ICOS signaling maintains eTreg cells, but not central Treg cell homeostasis, in both lymphoid and nonlymphoid tissue (39). In steady state conditions, ICOS is important for the accumulation of Treg cells in adipose tissue, but not in the lungs or skin (45). ICOS signaling has been shown to be important for induction of tolerance at mucosal sites (60, 61) as well as IL-10 production by Treg cells in certain models of inflammation (61–64). In our work, high levels of ICOS expression is well aligned with lung tissue resident Treg cell expansion and IL-10 production during flu, suggesting that ICOS^high^ is a feasible marker for characterizing active lung tissue resident Treg cells in flu models. Future studies may be able to further probe the function of these influenza-induced ICOS^high^ lung tissue resident Treg cells.

Knowledge of the role of ICOS and PD-1 signaling in lung tissue resident Treg cells is limited and has not been explored in the context of influenza infection. Our data found that expansion and IL-10 production by tissue resident Treg cells during influenza infection was counter-regulated by ICOS and PD-1 signaling. Opposing roles of ICOS and PD-1 signaling have been demonstrated for effector Treg cells, T follicular regulatory (T_fr_) and T follicular helper (T_fh_) cells in lymphoid tissues. ICOS and PD-1 signaling have been shown to counter-regulate effector Treg abundance at homeostasis in the spleen. ICOSL blockade reduces the numbers of effector Treg cells, while both PD-L1 blockade and Treg specific deletion of PD-1 results in effector Treg cell accumulation (39, 57). Following immunization, ICOS is required for the differentiation of T_fr_ cells while PD-1 inhibits T_fr_ differentiation in lymph nodes (LNs) and circulating in blood (65). In LN, ICOS and PD-1 signaling, *via* their antagonistic effects on PI3K signaling, counter-regulate T_fh_ recruitment to the follicle (66, 67). While it is tempting to speculate that PD-1 and ICOS regulate lung tissue resident Treg cell expansion and function *via* their opposing roles in PI3K/Akt signaling, there could be alternative explanations. PD-1 negatively regulates TCR/CD28 signaling and therefore multiple downstream pathways including PI3K/Akt, PKCθ, and ERK (68–71). In addition to PI3K/Akt, ICOS signals through TBK1 and promotes TCR induced calcium flux (72–74). It is also controversial whether PD-1 directly regulates ICOS signaling (69, 75, 76). Future studies utilizing Treg-specific deletions of PD-1 and ICOS, especially those allowing temporal control of the gene deletions, would aide in defining the roles of PD-1 and ICOS during different stages of flu and signaling pathways required for lung tissue resident Treg cell development and function.

There are several lines of evidence pointing towards a critical role of Treg cells during influenza infection. Depletion of Treg cells during primary influenza infection results in an increased effector CD4^+^ response (25), while depletion following clearance of the virus delays recovery and resolution of inflammation (77). The depletion of Treg cells prior to secondary influenza infection has been shown to increase inflammation, reduce lung function, and increase flu-specific CD8^+^ T cell response (55). Treg cell-derived amphiregulin has also been shown to be critical for preventing lung tissue damage during acute influenza infection, independent of suppressive function (26). The effects of Treg cell depletions are similar to those observed when IL-10 signaling was blocked in acute primary flu (78) and during resolution of primary flu infection (79). The role of effector T cell-derived IL-10 has been established in pulmonary immunomodulation during primary flu (78), while the role of Treg cell-derived IL-10 in host defense and immunopathology during primary and secondary influenza infections remains to be elucidated. Similar to our findings, anti-PD-L1 treatment increased the number of IL-10-producing Treg cells during *Toxoplasma gondii* infection, which coincided with the reduction of pathogen-specific effector T cell responses and can be reverted by blocking IL-10 receptor signaling (57), suggesting that a Treg cell-specific PD-1/IL-10 signaling axis is involved in host defense against infections. Mice with Treg cell-specific IL-10 deficiency revealed that IL-10 production by Treg cells was not required for the control of systemic autoimmunity, but it was essential in limiting immunopathology skewed by the environment at the mucosal barriers such as the colon and lungs (80). This model may be useful to help further define the role of Treg cell-derived IL-10 during influenza infections. However, to avoid artifacts due to spontaneous mucosal inflammation and allow time-specific deletion of Treg cell-derived IL-10, an inducible Treg cell-specific IL-10 deletion model would be highly desirable.

Taken together, using a unique Foxp3^RFP^/IL-10^GFP^ dual reporter mouse model and the gold-standard intravascular *in vivo* staining protocol for identifying lung tissue resident T cells, our work reported here provides a comprehensive characterization of lung tissue resident Treg cells during primary flu and flu re-infections. Our findings that PD-1 and ICOS signaling pathways counter-regulate lung tissue resident Treg cell expansion and IL-10 production during flu infections shed light on lung tissue resident Treg cell biology. The co-inhibitory and co-stimulatory signatures that are highly expressed by the lung tissue resident Treg cells, including PD-1, TIGIT, LAG-3, ICOS and GITR may be used as biomarkers to identify and evaluate the activities of lung tissue resident Treg cells during flu infections, while PD-1 and ICOS signaling may be used as molecular targets to modulate the numbers and functions of lung tissue resident Treg cells, and therefore, lung immune responses during acute pulmonary viral infections.

## Data availability statement

The raw data supporting the conclusions of this article are available upon request.

## Ethics statement

The animal study was reviewed and approved by the Institutional Animal Care and Use Committee at Louisiana State University.

## Author contributions

MCM and WH designed experiments, analyzed and interpreted data, and wrote the manuscript; MCM, TZ, NM, RI, MC, and WH performed experiments; WH conceived research, secured funding and supervised the research. All authors contributed to the article and approved the submitted version.

## Funding

This work was supported in part by grants from the National Institutes of Health (P20 GM130555-6610, R56 AI146226 and R01 AI151139 to WH). MCM is a fellow recipient of the Careers in Immunology Fellowship from the American Association of Immunologists. WH is a fellow recipient of the Research Publication Grant in Engineering, Medicine, and Science from the American Association of University Women.

## Acknowledgments

We thank the NIH Tetramer Core Facility (contract number 75N93020D00005) for providing influenza NP peptide loaded tetramers. We also thank Ms. Qingxia Wang for technical assistance, Drs. David Topham and Gary Whittaker for mouse adapted WSN and X31 viral stocks, and Drs. Jie Sun and Gus Kousoulas for advice and helpful discussions.

## Conflict of interest

WH received research support from MegaRobo Technologies Co., Ltd., which was not used in this study.

The remaining authors declare that the research was conducted in the absence of any commercial or financial relationships that could be construed as a potential conflict of interest.

## Publisher’s note

All claims expressed in this article are solely those of the authors and do not necessarily represent those of their affiliated organizations, or those of the publisher, the editors and the reviewers. Any product that may be evaluated in this article, or claim that may be made by its manufacturer, is not guaranteed or endorsed by the publisher.
