# Supplementary material for: PD-1 and ICOS counter-regulate tissue resident regulatory T cell development and IL-10 production during flu
Source: Front Immunol. 2022 Sep 8;13:984476. doi: 10.3389/fimmu.2022.984476 (PMC9492985; doi:10.3389/fimmu.2022.984476)
Supplement: Supplementary file 1 [file Presentation_1.pdf]

Supplementary Materials

PD-1 and ICOS counter-regulate tissue resident regulatory T cell development and IL-10 production during flu

Michael C. McGee[1], Tianyi Zhang[1], Nicholas Magazine[1], Rezwanul Islam[1], Mariano Carossino[1], Weishan Huang[1,2,*]

[1]Department of Pathobiological Sciences, School of Veterinary Medicine, Louisiana State University, Baton Rouge, LA, USA
[2]Department of Microbiology and Immunology, College of Veterinary Medicine, Cornell University, Ithaca, NY, USA

[*] Corresponding author:
Weishan Huang, PhD:
Address: 1909 Skip Bertman Drive, Baton Rouge, LA 70803, USA
Tel.: +01-225-5789467; Fax: +01-225-5789701; e-mail: huang1@lsu.edu

Running title: Tissue resident Treg cells during flu

Key words:
Influenza infections, tissue resident T cells, Foxp3, ICOS, PD-1, IL-10

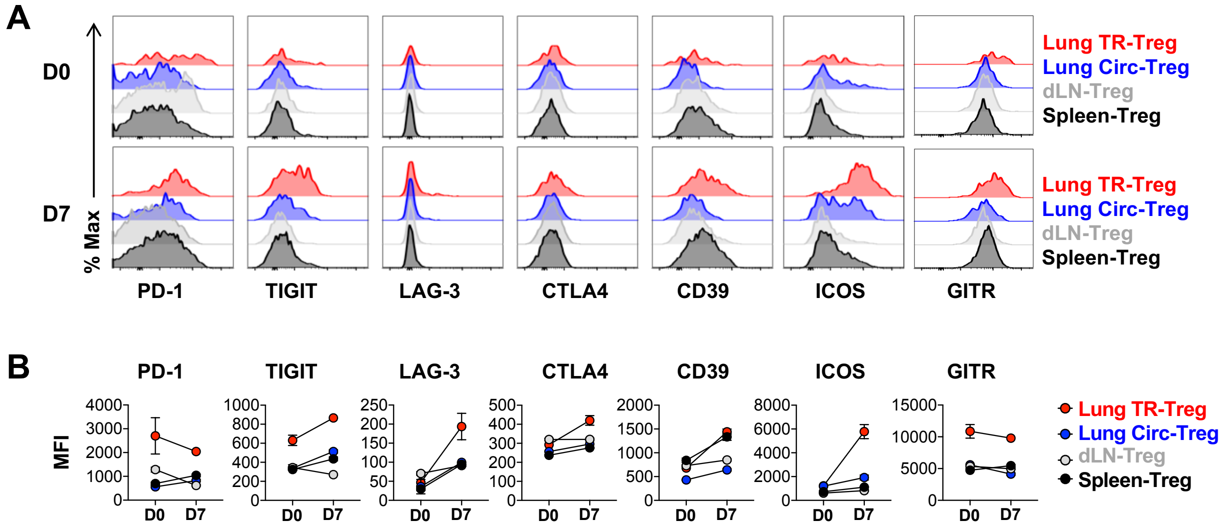

Supplemental Figure 1: Characterization of functional marker expression by lung TR-Treg cells following influenza infection.

Lung TR-Treg, lung Circ-Treg, dLN-Treg, and spleen-Treg cells from Figure 2A were also analyzed for the levels of expression of multiple immune co-inhibitory and co-stimulatory molecules.

(A) Representative flow histograms of PD-1, TIGIT, LAG-3, CTLA4, CD39, ICOS, and GITR.

(B) Summary of MFI values of PD-1, TIGIT, LAG-3, CTLA4, CD39, ICOS, and GITR.

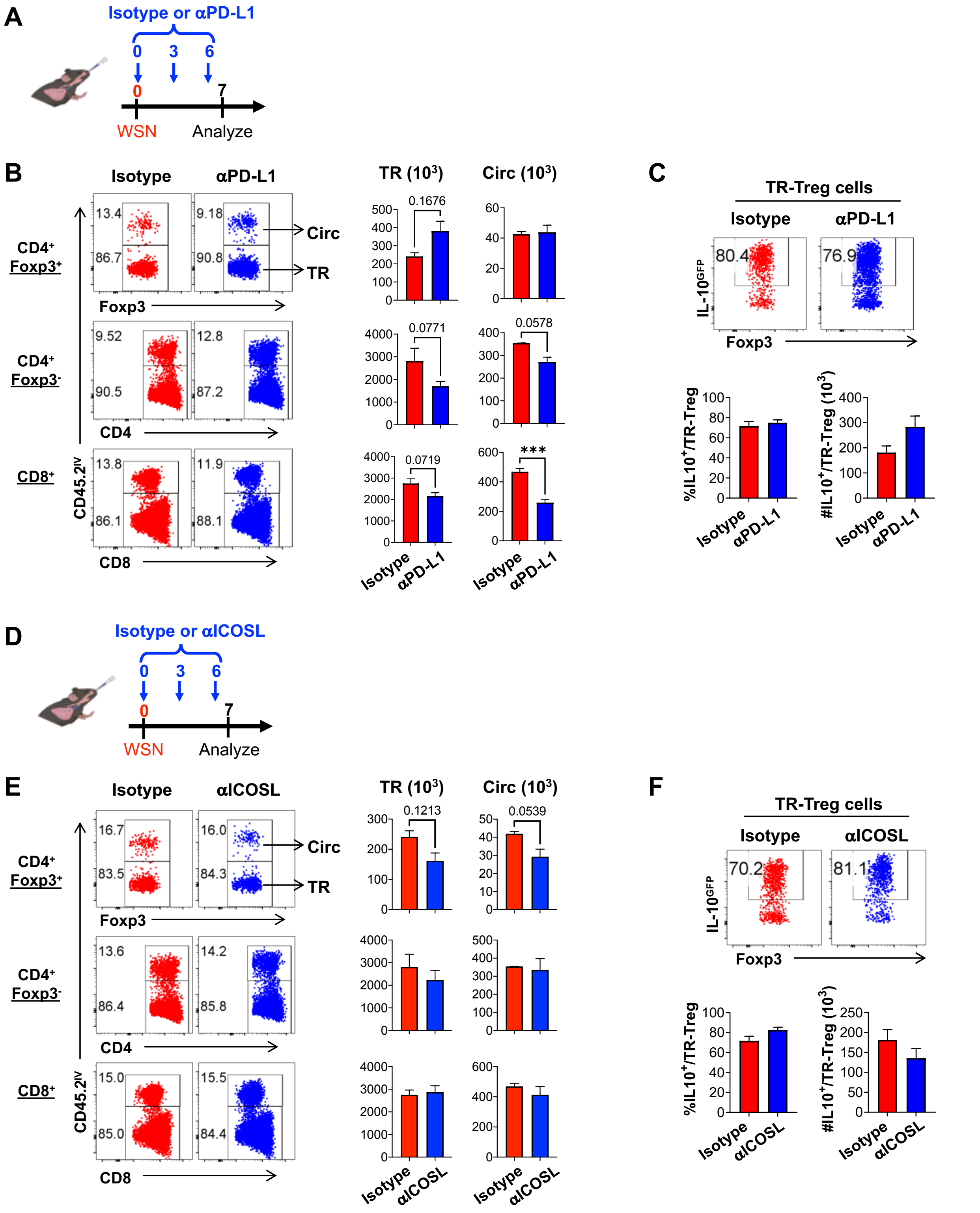

Supplemental Figure 2: PD-1 and ICOS signaling does not significantly impact lung tissue resident Treg cell expansion or IL-10 production during acute primary influenza infection.

(A-C) PD-1 signaling blockade slightly increases lung tissue resident Treg cell expansion but does not affect IL-10 production during acute primary influenza infection.
(A) Schematics of PD-1 signaling blockade during primary influenza infection: Foxp3^{RFP}/IL-10^{GFP} dual reporter mice were infected with 10^3 PFU of WSN virus (H1N1, IN). The date of primary infection was set as "D0". Anti-PD-L1 antibody (or isotype control) was injected intravenously on D0, D3 and D6, while mice were analyzed on D7.
(B) Representative flow plots of *in vivo* staining of CD45.2 and summary of numbers of TR versus Circ CD4$^+$ Foxp3$^+$ (Treg), CD4$^+$ Foxp3$^-$, and CD8$^+$ T cells isolated from the lungs of the isotype or anti-PD-L1 antibody-treated mice.
(C) Representative flow plots of IL-10^{GFP} expression by lung TR-Treg cells, in mice receiving isotype or anti-PD-L1 treatment during primary influenza infection. Summary of the percentage of IL-10$^+$ fraction and numbers of IL-10$^+$ lung TR-Treg cells under the indicated treatment conditions were shown as well.
(D-F) ICOS signaling blockade slightly attenuates lung tissue resident Treg cells expansion but does not affect IL-10 production during acute primary influenza infection.
(D) Schematics of ICOS signaling blockade during primary influenza infection: Foxp3^{RFP}/IL-10^{GFP} dual reporter mice were infected as indicated in (A). The date of secondary infection was set as "D0". Anti-ICOSL antibody (or isotype control) was injected intravenously on D0, D3 and D6, while mice were analyzed on D7.
(E) Representative flow plots of *in vivo* staining of CD45.2 and summary of numbers of TR versus Circ CD4$^+$ Foxp3$^+$ (Treg), CD4$^+$ Foxp3$^-$, and CD8$^+$ T cells isolated from the lungs of the isotype or anti-ICOSL antibody-treated mice.
(F) Representative flow plots of IL-10^{GFP} expression by lung TR-Treg cells, in mice receiving isotype or anti-ICOSL treatment during primary influenza infection. Summary of the percentage of IL-10$^+$ fraction and numbers of IL-10$^+$ lung TR-Treg cells under the indicated treatment conditions were shown as well.
$N \geq 3$. Data were combined from two different experiments. *P* values were calculated by unpaired student *t* test. Data presented as Mean ± S.E.M..

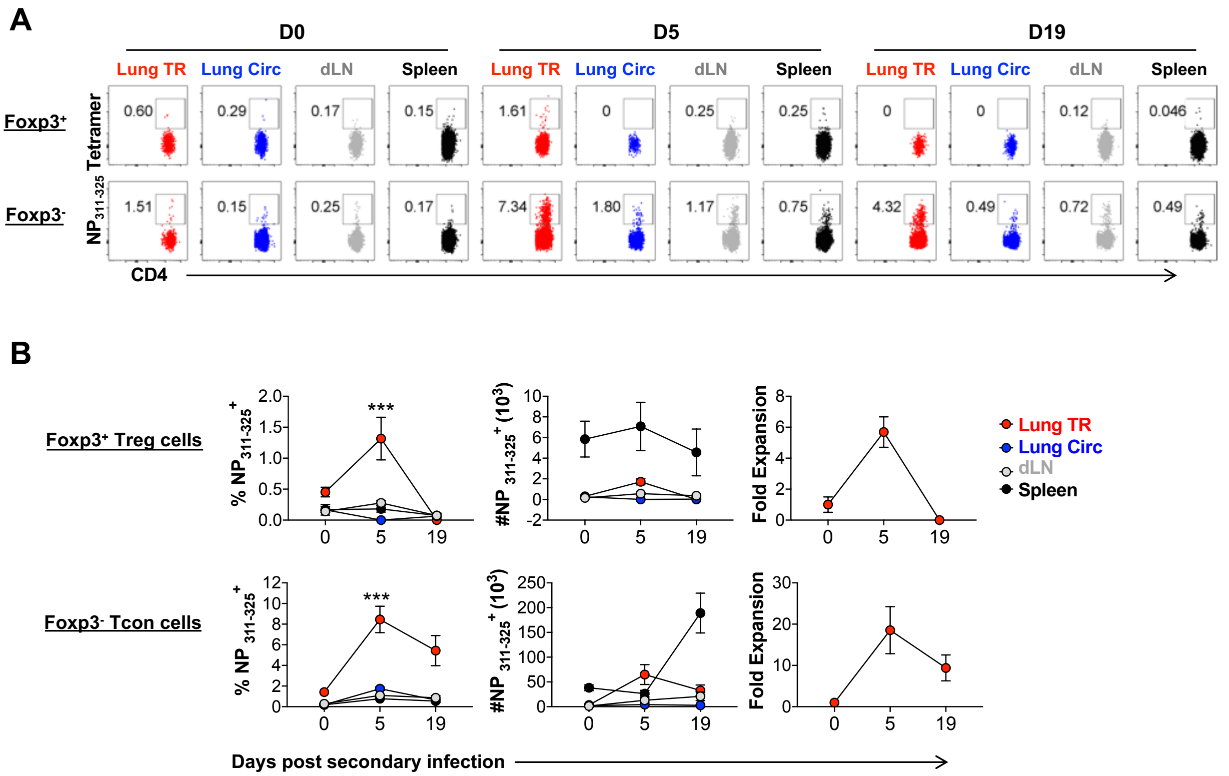

Supplemental Figure 3: Kinetics of influenza $NP_{311-325}$ specific $CD4^+$ T cells during secondary influenza infection.

T cells from mice treated as shown in Figure 5A were further analyzed for influenza A $NP_{311-325}$ loaded MHCII tetramer binding.

(A) Representative flow plots of $NP_{311-325}$ tetramer bound $Foxp3^+$ versus $Foxp3^-$ $CD4^+$ T cell subsets from different compartments: lung tissue residents, lung circulating cells, dLN, and spleen, isolated prior to (D0), and 5 (D5) and 19 (D19) days post secondary infection.

(B) Summary of percentages and numbers of $NP_{311-325}$ tetramer bound cells in $Foxp3^+$ versus $Foxp3^-$ $CD4^+$ T cell subsets from different compartments: lung TR, lung Circ, dLN, and spleen, on D0, D5, D19 during secondary influenza infection. Fold expansion of lung tissue resident $Foxp3^+$ (Treg) versus $Foxp3^-$ (Tcon) $CD4^+$ cells is shown as well; the average number of cells on D0 was set as "1" for fold change calculation. $N \geq 3$. $^{***}p \leq 0.001$, by two-way ANOVA with post-hoc tests for time point-pairwise comparisons. Data presented as Mean ± S.E.M..

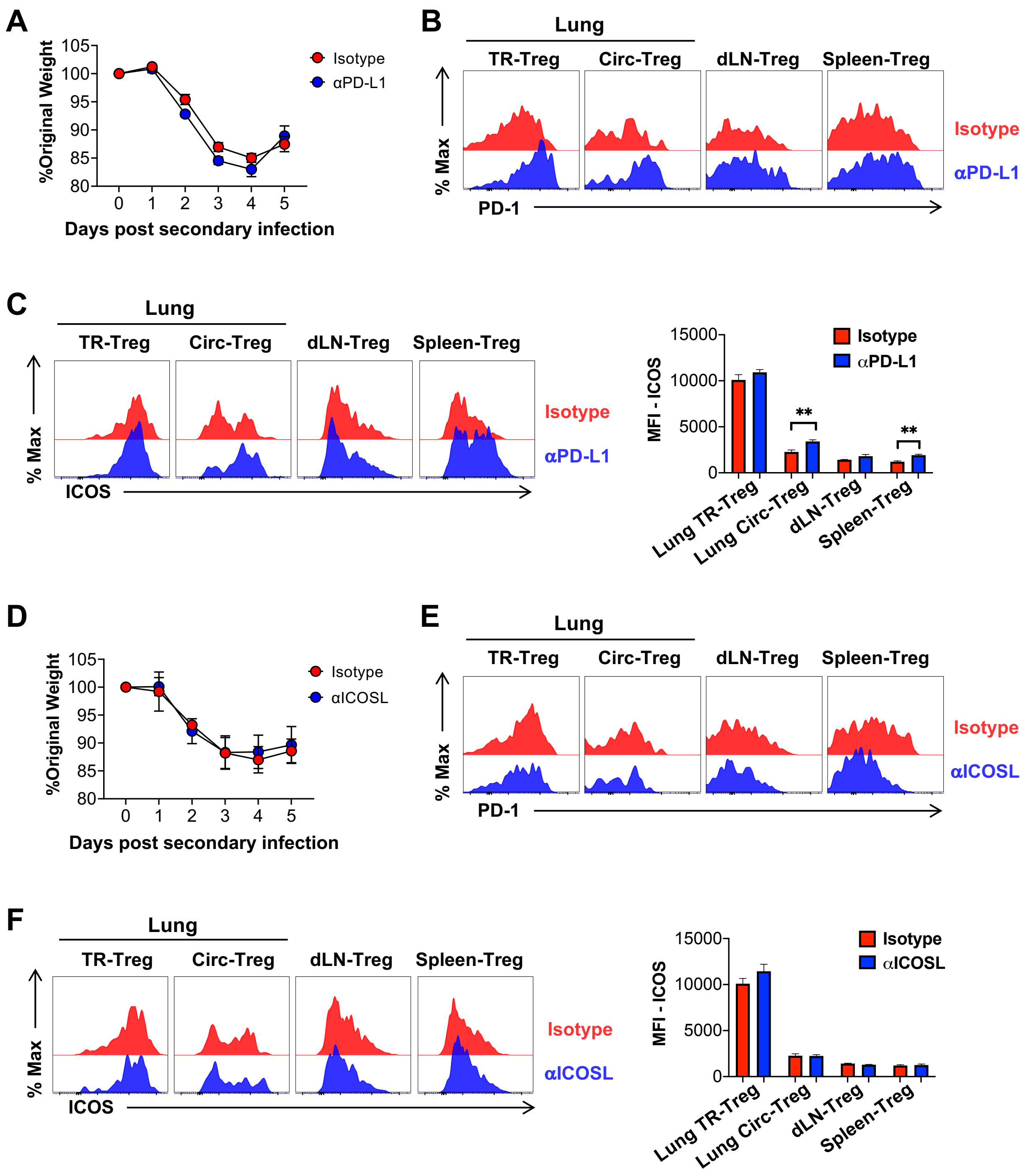

Supplemental Figure 4: Blockade of PD-1 or ICOS signaling regulates PD-1 but not ICOS expression during secondary influenza infection.

Animals and cells from Figure 7 were analyzed further.

(A) Percentage of the original weight of isotype and anti-PD-L1 treated mice following secondary influenza infection.

(B) Representative plots of PD-1 expression by lung TR-Treg, lung Circ-Treg, dLN-Treg, and spleen-Treg cells, isolated from mice that received either the isotype or anti-PD-L1 antibody treatment. Corresponding plots to data summarized in Figure 7B.
(C) Representative plots and summary of ICOS expression (MFI) by lung TR-Treg, lung Circ-Treg, dLN-Treg, and spleen-Treg cells, isolated from mice that received either the isotype or anti-PD-L1 antibody treatment.
(D) Percentage of the original weight of isotype and αICOSL treated mice following secondary influenza infection.
(E) Representative plots of PD-1 expression by lung TR-Treg, lung Circ-Treg, dLN-Treg, and spleen-Treg cells, isolated from mice that received either the isotype or anti-ICOSL antibody treatment. Corresponding plots to data summarized in Figure 7E.
(F) Representative plots and summary of ICOS expression (MFI) by lung TR-Treg, lung Circ-Treg, dLN-Treg, and spleen-Treg cells, isolated from mice that received either the isotype or anti-ICOSL antibody treatment.
N ≥ 3. Data represent the results of at least two different experiments and are presented as Mean ± S.E.M..

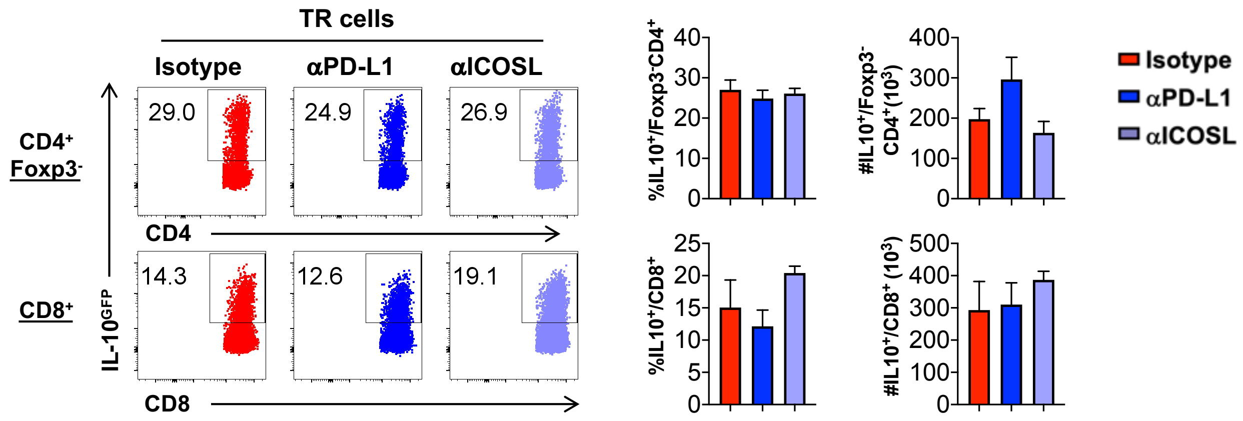

Supplemental Figure 5: Blockade of PD-1 or ICOS signaling does not affect IL-10 production in tissue resident conventional T cells during secondary influenza infection.
Representative flow plots of IL-10^{GFP} expression by lung tissue resident conventional $CD4^+$ and $CD8^+$ T cells, in mice receiving isotype, anti-PD-L1 or anti-ICOSL treatment during secondary influenza infection, as shown in Figure 7. Summary of the percentage of IL-10^+ fraction and numbers of IL-10^+ lung tissue resident conventional $CD4^+$ and $CD8^+$ T cells under the indicated treatment conditions were shown as well. N $\geqslant$ 3. Data were combined from three different experiments and presented as Mean ± S.E.M..
